# Embracing Diversity: Differences in Virulence Mechanisms, Disease Severity, and Host Adaptations Contribute to the Success of Nontyphoidal *Salmonella* as a Foodborne Pathogen

**DOI:** 10.3389/fmicb.2019.01368

**Published:** 2019-06-26

**Authors:** Rachel A. Cheng, Colleen R. Eade, Martin Wiedmann

**Affiliations:** ^1^Department of Food Science, Cornell University, Ithaca, NY, United States; ^2^Department of Population Medicine and Diagnostic Sciences, Cornell University, Ithaca, NY, United States; ^3^Department of Chemistry, University of North Carolina at Charlotte, Charlotte, NC, United States

**Keywords:** nontyphoidal *Salmonella*, virulence, serovars, foodborne pathogen, food safety

## Abstract

Not all *Salmonella enterica* serovars cause the same disease. *S. enterica* represents an incredibly diverse species comprising >2,600 unique serovars. While some *S. enterica* serovars are host-restricted, others infect a wide range of hosts. The diseases that nontyphoidal *Salmonella* (NTS) serovars cause vary considerably, with some serovars being significantly more likely to cause invasive disease in humans than others. Furthermore, while genomic analyses have advanced our understanding of the genetic diversity of these serovars, they have not been able to fully account for the observed clinical differences. One overarching challenge is that much of what is known about *Salmonella*’s general biology and virulence strategies is concluded from studies examining a select few serovars, especially serovar Typhimurium. As targeted control strategies have been implemented to control select serovars, an increasing number of foodborne outbreaks involving serovars that are less frequently associated with human clinical illness are being detected. Harnessing what is known about the diversity of NTS serovars represents an important factor in achieving the ultimate goal of reducing salmonellosis-associated morbidity and mortality worldwide. In this review we summarize the current understanding of the differences and similarities among NTS serovars, highlighting the virulence mechanisms, genetic differences, and sources that characterize *S. enterica* diversity and contribute to its success as a foodborne pathogen.

## Introduction

Salmonellae are Gram-negative, facultatively anaerobic bacteria. The genus *Salmonella*, named for Dr. Daniel Salmon, was first described in 1866 by Dr. Theobald Smith (Schultz, 2008). Initially, *Salmonella* was described as the causative agent of pig cholera (first named *Salmonella choleraesuis*). However, pig cholera was later discovered to be a viral disease, with *Salmonella* co-infection being common (Schultz, 2008).

**FIGURE 1:**
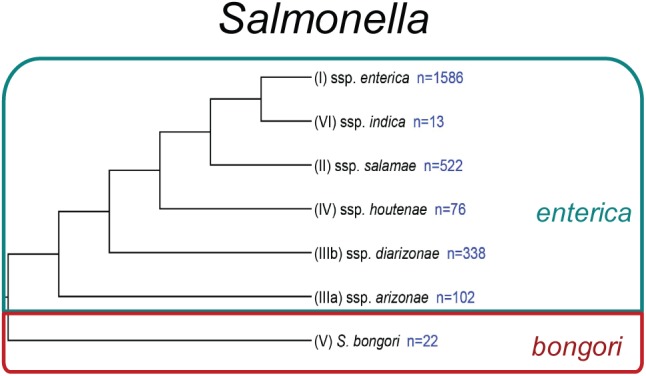
Phylogeny of *Salmonella*. Phylogenetic analysis reconstructed from Desai et al. (2013). Subspecies identifiers preceding the subspecies (ssp.) name are shown in parentheses. The number of currently recognized serovars (Issenhuth-Jeanjean et al., 2014) within each species or subspecies is listed in blue.

Today, the genus *Salmonella* includes 2 species: *enterica* and *bongori* (Brenner et al., 2000; Figure 1). Within the species *enterica*, there are 6 subspecies, which are characterized by both a name and a roman numeral: *enterica* (I), *salamae* (II), *arizonae* (IIIa), *diarizonae* (IIIb), *houtenae* (IV), and *indica* (VI) (Brenner et al., 2000). *Salmonella bongori* was originally classified as *Salmonella enterica* subspecies V, before being re-classified as a separate species (Brenner et al., 2000). A third species, *Salmonella subterranean* was proposed (Shelobolina et al., 2004), however, further DNA characterization revealed that this species did not belong in the genus *Salmonella* (Issenhuth-Jeanjean et al., 2014). Recent analyses suggest there are four major phylogenetic clades within *S. enterica* subsp. *enterica* (Worley et al., 2018). The majority of *Salmonella* isolates infecting warm-blooded hosts belong to subspecies *enterica* (I), while subspecies II-VI and *S. bongori* are primarily isolated from environmental sources or cold-blooded hosts, such as reptiles and amphibians.

Infection with typhoidal (*S. enterica* subsp. *enterica* serovar Typhi) and paratyphoidal serovars (i.e., serovars Paratyphi A, Paratyphi B, Paratyphi C, and Sendai) typically results in an invasive, extra-intestinal disease characterized by a high fever (>39°C), malaise, vomiting, headache, and an elevated pulse rate (Dougan and Baker, 2014; Hiyoshi et al., 2018). Serovar Typhi is host-restricted, and is transmitted human-to-human primarily via fecal contamination of drinking water or improper food handling (Connor and Schwartz, 2005). In contrast, infections with NTS serovars (i.e., all serovars except Typhi, Paratyphi A, Paratyphi B, Paratyphi C, or Sendai) typically result in a self-limiting gastroenteritis that is cleared by the host within 4–7 days (Gal-Mor et al., 2014). Due to these stark discrepancies in disease manifestation, nontyphoidal and typhoid salmonellosis cases are frequently assessed and reported independently.

Serovar Typhimurium is the best-studied serovar for nontyphoidal salmonellosis and has become the “model serovar” for studying NTS (Sabbagh et al., 2010; Tsolis et al., 2011; Fàbrega and Vila, 2013; Rivera-Chavez and Baumler, 2015). The *S*. Typhimurium strain LT2 has been widely used since the 1940s and has been characterized extensively (Swords et al., 1997). In fact many studies have used LT2 as a model for NTS, despite the fact that this strain encodes a rare start codon (UUG) in its *rpoS*, rendering this strain avirulent (Lee et al., 1995; Wilmes-Riesenberg et al., 1997). This suggests that conclusions drawn from experiments performed with *S*. Typhimurium LT2 should be cautiously considered, as this lab strain may not accurately represent NTS virulence. Furthermore, NTS vary significantly with respect to the severity of the illness that they cause, as infection with certain serovars is significantly more likely to result in human invasive disease, hospitalization, and death when compared to the rates of invasive disease observed for *S*. Typhimurium (Jones et al., 2008). This suggests that while *S*. Typhimurium was useful as a general model for NTS, there are likely additional virulence factors, adaptations, and other fitness determinants that may not be accurately represented in *S*. Typhimurium.

**FIGURE 2:**
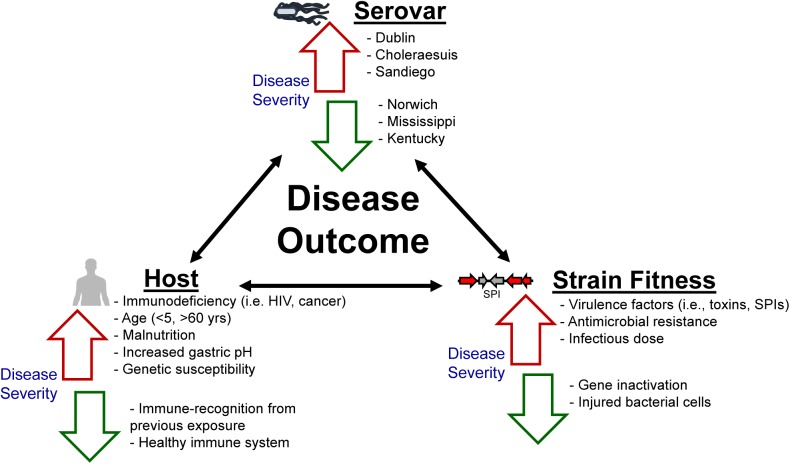
Not all nontyphoidal salmonellosis infections are alike. The morbidity and mortality associated with nontyphoidal salmonellosis exists as an interplay between the host’s immune response as well as the overall fitness of the serovar/strain. Factors listed adjacent to red arrows have been previously established as contributing to increased disease severity, whereas factors listed adjacent to green arrows are associated with a decrease in disease severity.

Despite extensive government and industry efforts aimed at reducing the incidence of nontyphoidal salmonellosis, little progress has been made in reducing the number of salmonellosis cases per year (Boore et al., 2015; Ford et al., 2016). The various hosts that NTS serovars encounter and colonize demonstrate their tremendous adaptability. Differences in food consumption, sanitation, cultural traditions, infrastructure, and food safety regulations all influence the global burden of nontyphoidal salmonellosis. Furthermore, differences in host susceptibility, virulence factors/mechanisms specific to select serovars, as well as serovar fitness, contribute to the severity, and outcome of salmonellosis (Figure 2). The role of antimicrobial resistance in the expansion of select NTS subtypes in a novel niche, as well as the potential implications associated with increasing the severity of the outcome of infection due to rapid outgrowth of an antibiotic-resistant *Salmonella* strain in the GI tract of antibiotic-treated individuals, have been reviewed extensively elsewhere (Rabsch et al., 2001; Furuya and Lowy, 2006; Crump et al., 2015), and therefore will not be covered in this review.

A number of reviews have detailed the host-response to both nontyphoidal and typhoid salmonellosis (Gal-Mor et al., 2014; Gilchrist et al., 2015; Keestra-Gounder et al., 2015; LaRock et al., 2015; Rivera-Chavez and Baumler, 2015). However, a better understanding of the diversity of NTS serovars and which adaptations make them more suited to colonize different hosts or survive in different environments, is lacking. In this review, we have synthesized studies representing the diversity of NTS serovars to demonstrate how *Salmonella* uses a variety of virulence factors, and genetic and phenotypic adaptations to become one of the most successful foodborne pathogens worldwide.

### Classical and Modern Approaches to Defining and Studying NTS Diversity

**FIGURE 3:**
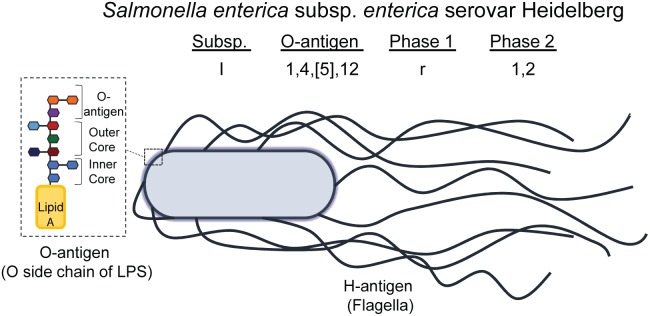
Illustration of NTS O- and H-antigens, comprising the serotyping scheme defined by Kauffman-White-Le Minor. The antigenic formula of a serovar is composed of the subspecies (I -VI), the O-antigen (numbered), and the flagellar antigens called phase 1, phase 2, and phase 3 (if present). Some serovars have lost their phase 1 and/or phase 2 antigen and others have gained additional flagellar antigens (phase 3). The O-antigen illustrated represents the terminal side chain of lipopolysaccharide (LPS) on the cell wall of Gram-negative bacteria, such as *Salmonella*, which is composed of an inner and outer core of primarily polysaccharides bound to Lipid A; the O side chain is composed of repeating saccharide units, and is the subunit of LPS that differs between serogroups. *Salmonella enterica* subsp. *enterica* serovar Heidelberg, the NTS serovar modeled here, is the common name of the serovar having antigenic formula I 1,4,[5],12:r:1,2.

Salmonellae are differentiated based on the reactivity of the O (somatic, or O polysaccharide component of lipopolysaccharide [LPS]) and H (flagellar) antigens to anti-sera, in a classification scheme known as the Kauffman-White-Le Minor scheme (Figure 3; Brenner et al., 2000; Liu et al., 2014). Salmonellae are categorized into 46 unique O groups (Liu et al., 2014), with 114 unique H antigens (McQuiston et al., 2004). Together with the roman numeral abbreviation for the *S. enterica* subspecies, the combination of O- and H-antigens antigens is what defines the serotype (also known as a serovar) of a given *Salmonella* strain. *Salmonella* serovars also have common names that are often the name of the geographical location where they were first isolated (Gossner et al., 2016), to accompany their antigenic formulae (Brenner et al., 2000).

Another traditional classification scheme, called phage typing, involves subjecting *Salmonella* strains to libraries of phage to determine which phage are able to lyse a given strain. Examples of historically important phage types include *S*. Typhimurium DT (definitive type) 104, which arose as an important multi-drug resistant (MDR) strain in the United Kingdom in the early 1960s that subsequently spread and became a global epidemic (Threlfall, 2000), and *S*. Enteritidis PT (phage type) 4, which is associated with contamination of intact chicken eggs (Humphrey, 1994).

Although modern genetic analyses are now typically used to predict the phage type of a given *Salmonella* isolate, phage typing may still be reported as an additional level of discrimination. Likewise, other *in silico* typing schemes have increased in popularity, as they provide increased discriminatory power and negate the use for traditional serotyping. Rather, these methods utilize serotype prediction based on sequence typing of the O- and H-antigens (McQuiston et al., 2004), or more recently, using whole genome sequence (WGS) data (Zhang et al., 2015; Yoshida et al., 2016; Robertson et al., 2018). The sequence data are compared to a database of sequences, which compare the O- and H-antigens of known serotypes to predict which anti-sera an isolate would react with, and therefore, the serotype of the isolate. Other identification schemes are based on sequence comparisons of housekeeping genes (Achtman et al., 2012), and more recently on core genome or whole genome multi-locus sequence typing (Alikhan et al., 2018).

Recent phylogenetic analyses have shed light on the fact that many commonly isolated serovars (serovars Newport, Montevideo, Kentucky, Paratyphi B, Derby, Nchanga, Cerro, Bareilly, Stanleyville, Dusseldorf, Livingstone, and others) are polyphyletic (Den Bakker et al., 2011; Cao et al., 2013; Timme et al., 2013; Yoshida et al., 2016; Sévellec et al., 2018; Worley et al., 2018). In fact, a recent study comparing 266 different serovars reported that ∼10% are polyphyletic or paraphyletic (Worley et al., 2018). The polyphyletic nature of some serovars may provide further epidemiological evidence that can assist in outbreak investigations by providing additional discriminatory power, as was recently proposed for *S. enterica* subsp. *enterica* serovar Derby among different regions in France (Sévellec et al., 2018). WGS subtyping adds an additional level of discriminatory power that can be used to aid epidemiologic investigations of traceback studies. There are also important clinical implications of polyphyletic *Salmonella* serovars that arise when different clades of the same serovar differ in the virulence factors that they encode, as is the case for *S*. Mississippi, where one clade encodes typhoid toxin genes, and the other does not (Miller and Wiedmann, 2016). Genomic analyses are just beginning to identify polyphyletic serovars, and to define key differences associated with the different clinical outcomes observed.

### Geographic Diversity – Distribution of NTS Serovars Is Regionally Associated

Infections with NTS account for just over one fifth of all bacterial foodborne illnesses worldwide, causing an estimated 78.7 million cases per year (Havelaar et al., 2015). In the United States NTS is the leading cause of bacterial foodborne illness, resulting in an estimated 1.2 million illnesses, 23,128 hospitalizations, and 452 deaths annually (Scallan et al., 2011). In the US, the 20 NTS serovars most commonly isolated from human clinical cases account for nearly 70% of all NTS clinical cases in humans (Centers for Disease Control and Prevention [CDC], 2016). According to WHO estimates of foodborne disease, the global incidence of nontyphoidal salmonellosis as of 2010 was 1,140 cases per 100,000 people (1.14% of people) (Kirk et al., 2015). However, the burden of nontyphoidal salmonellosis is not equally distributed among different geographic regions. Countries in the Eastern Mediterranean region (e.g., Iran, Kuwait, Saudi Arabia, Egypt, and others) had the highest estimated incidence of nontyphoidal salmonellosis (1,610 cases per 100,000), while the European region (e.g., European Union, Russia, Ukraine, Switzerland, and others) had the lowest (186 cases per 100,000) (Kirk et al., 2015). However, the African region had the highest death rate from nontyphoidal salmonellosis: 1 death per 100,000 persons compared to a global rate of 0.4 deaths per 100,000 persons (Kirk et al., 2015). In contrast to other foodborne bacterial pathogens such as *E. coli* and *Campylobacter* spp., the majority of NTS salmonellosis occurs in individuals above 5 years of age (Havelaar et al., 2015).

**FIGURE 4:**
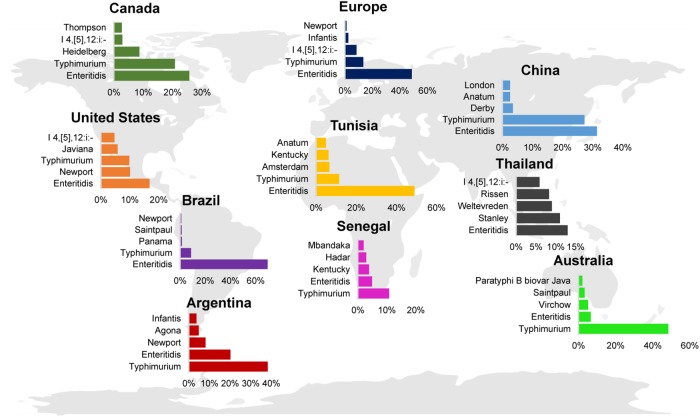
Global distribution of the top 5 NTS serovars associated with human clinical disease. Bar charts represent the top 5 NTS (i.e., serovar Typhi was excluded) serovars reported for Argentina (2007) (Hendriksen et al., 2011), Australia (2011) (OzFoodNet Working Group, 2015), Brazil (2007) (Hendriksen et al., 2011), Canada (2007) (Hendriksen et al., 2011), China (2008) (Ran et al., 2011), the European Union (2016) (European Food Safety Authority [EFSA] and European Centre for Disease Prevention, and Control [ECDC], 2017), Senegal (2007) (Hendriksen et al., 2011), Thailand (2004) (Hendriksen et al., 2009), Tunisia (2007) (Hendriksen et al., 2011), and the United States (2016) (Centers for Disease Control and Prevention [CDC], 2016). Only human clinical cases are reported; data were reported from 2004 to 2016.

Despite the discrepancy in rates of illness per geographic region, Typhimurium and Enteritidis are consistently reported as the serovars contributing the highest proportion of human clinical salmonellosis worldwide (Figure 4; Hendriksen et al., 2009, 2011; Ran et al., 2011; OzFoodNet Working Group, 2015; Centers for Disease Control and Prevention [CDC], 2016; European Food Safety Authority [EFSA] and European Centre for Disease Prevention, and Control [ECDC], 2017). Interestingly, in some countries the proportion of human clinical cases are dominated by one serovar, such as Typhimurium in Australia or Enteritidis in Brazil, while other countries show a more balanced distribution of cases per serovar (e.g., Enteritidis, Newport, and Typhimurium in the United States). The serovars representing the 3rd, 4th, and 5th most commonly isolated serovars from human clinical infections appear to be more geographically associated. For example, serovars that are found among human cases in Australia (serovars Saintpaul and Virchow) are less common in other countries (Figure 4).

The regional differences observed among the incidence of infection by select serovars may be explained by differences in (i) animal hosts that populate the region, (ii) the quality of surveillance and reporting systems (Kirk et al., 2015), which may effectively underestimate the incidence of some serovars, (iii) concurrent immunodeficiencies such as HIV infection or cancer (Okoro et al., 2012), (iv) dietary intake, (v) farming practices or food production practices that might select for specific serovars, or (vi) environmental factors influencing the cultivation, survival, or routes of transmission. In support of differences in environmental exposures, within the United States some serovars are regionally distributed, with some serovars representing the majority of clinical isolates in some geographical regions, but being rarely isolated in other regions (e.g., *S*. Mississippi) (Boore et al., 2015). This suggests that the majority of human clinical NTS salmonellosis cases in the United States are the result of contamination events that happen at a local level, given that food is often distributed across the country, if not internationally.

## Diversity of NTS Virulence Factors and Their Implications in Disease Manifestation

Reflective of an extensively host-adapted lifestyle is the collection of virulence factors possessed by NTS. These include flagella, fimbriae, toxins, pathogenicity islands, and virulence-associated plasmids. These features do not occur in all NTS serovars, and thus their presence or absence influences the virulence and host range of a particular isolate or serovar.

### *Salmonella* Pathogenicity Islands (SPIs)

To date, 24 *Salmonella* pathogenicity islands have been identified. These horizontally acquired loci encode genes facilitating several virulence mechanisms, including (i) the expression of secretion systems, fimbriae, flagella, and capsules, (ii) serotype conversion, and (iii) host colonization and subsequent survival within the host (Van Asten and Van Dijk, 2005; Fàbrega and Vila, 2013).

*Salmonella* Pathogenicity Islands 1 and 2 are the best characterized in terms of genetic and phenotypic traits. Of the 24 SPIs (Table 1), SPI-1 is ubiquitous among all *Salmonella* species and subspecies (Fookes et al., 2011). SPI-1 encodes a type three secretion system (T3SS), which is essential for export of effector proteins required for invasion of host cells. In contrast, SPI-2 is found only in *S. enterica*, and SPI-22 is only found in *S. bongori* (Fookes et al., 2011). SPI-2 encodes an additional T3SS, harboring genes that are essential for intracellular survival and for preventing acidification of the *Salmonella* containing vacuole (SCV). The remaining 21 SPIs are variably present among *S. enterica* (Kingsley et al., 2003; Shah et al., 2005; Boyen et al., 2006; Saroj et al., 2008; Blondel et al., 2009, 2013; Den Bakker et al., 2011; Shah et al., 2012; Hayward et al., 2013; Lee et al., 2013; Tomljenovic-Berube et al., 2013), with all but three having been found in NTS serovars; SPI-20 and -21 have been found only in *S. enterica* subsp. *arizonae* (Blondel et al., 2009), and while SPI-15 occurs in *S. enterica* subsp. *enterica*, it has only been identified in *S*. Typhi to date (Vernikos and Parkhill, 2006; Sabbagh et al., 2010).

**Table 1:** Current understanding of the size, function, and distribution of *Salmonella* pathogenicity islands (SPIs) among *Salmonella.*

SPI^1^	Size/location^2^ (kb)	Main genes or gene products^3^	Proposed function	Relative distribution^4,5^	References
SPI-1	40	T3SS, effector proteins (e.g., *avrA, iacB, invB, sicA, sicP, sipA, sipB, sipC*, and *sptP*)	Host cell invasion and enteropathy	*S. enterica S. bongori*	Fookes et al., 2011; Cao et al., 2013; Fàbrega and Vila, 2013
SPI-2	40	T3SS, effector proteins (*ssaB, ssaE, sscA, sscB, sseA, sseF, sseG*, and *ttr* genes)	Intracellular survival	*S. enterica* subsp. *enterica* *S. enterica* subsp. *salamae* *S. enterica* subsp. *diarizonae* *S. enterica* subsp. *indica*	Fookes et al., 2011; Fàbrega and Vila, 2013
SPI-3	Variable (17–36)	Magnesium transport system (*mgtCB*), *misL*	Intracellular survival, intestinal colonization	*S. enterica* subsp. *enterica* *S. enterica* subsp. *salamae* *S. enterica* subsp. *arizonae* (partial) *S. enterica* subsp. *diarizonae* (partial) *S. enterica* subsp. *houtenae* (partial) *S. enterica* subsp. *indica S. bongori *	Dorsey et al., 2005; Sabbagh et al., 2010; Fookes et al., 2011; Kaur and Jain, 2012
SPI-4	25	T1SS (*siiABCDF*), non-fimbrial adhesin (*siiE*)	Adhesion and invasion of epithelial cells, virulence in mice and cows	*S. enterica* subsp. *enterica S. enterica* subsp. *houtenae* *S. enterica* subsp. *indica S. bongori *	Morgan et al., 2007; Sabbagh et al., 2010; Fookes et al., 2011
SPI-5	Variable (11–44)	Effectors of SPI-1 and -2 (*pipABC, sopB, pipD*, and *sigDE*)	Epithelial invasion, enteric salmonellosis, and chicken colonization	*S. enterica* subsp. *enterica *	Fookes et al., 2011; Shah et al., 2012; Fàbrega and Vila, 2013; Cao et al., 2014
SPI-6	Variable (47–59)	T6SS, atypical fimbriae (*safABCD*), fimbriae (*tcfABCD*), and *sciS*	Invasion, intramacrophage survival, chicken colonization, and virulence in mice	*S. enterica* subsp. *enterica* *S. enterica* subsp. *salamae* (partial) *S. enterica* subsp. *arizonae* (partial) *S. enterica* subsp. *diarizonae* (partial) *S. enterica* subsp. *houtenae* (partial) *S. enterica* subsp. *indica* (partial)	Parkhill et al., 2001; Fookes et al., 2011; Cao et al., 2014; Pezoa et al., 2014
SPI-7	134	Vi capsule biosynthesis genes, SopE prophage, and type IVb pilus	Vi exopolysaccharide, host immune modulation, and intramacrophage survival	*S. enterica* subsp. *enterica* serovars Typhi, Paratyphi C, Dublin	Pickard et al., 2003; Faucher et al., 2005; Fookes et al., 2011
SPI-8	6.8	Bacteriocin fragment	Unknown	*S. enterica* subsp. *enterica*	Faucher et al., 2005; Saroj et al., 2008; Sabbagh et al., 2010; Fookes et al., 2011; Desai et al., 2013
SPI-9	16	T1SS, adhesin	Transport, epithelial adherence	*S. enterica* subsp. *enterica* *S. enterica* subsp. *salamae* *S. enterica* subsp. *arizonae* *S. enterica* subsp. *diarizonae* *S. enterica* subsp. *houtenae* *S. enterica* subsp. *indica* *S. bongori*	Sabbagh et al., 2010; Fookes et al., 2011; Velasquez et al., 2016
SPI-10	33	P4-like prophage, *Sef* fimbriae	Virulence in mice and chickens, intramacrophage uptake or survival	*S. enterica* subsp. *enterica*	Parkhill et al., 2001; Bishop et al., 2005; Sabbagh et al., 2010
SPI-11	Variable (6–10)	*pagCD, envF* (some) sRNA RaoN, typhoid toxin gene islet (*cdtB, pltA*, and *pltB*) (some)	Intramacrophage survival, serum resistance, and typhoid fever pathology	*S. enterica* subsp. *enterica*	Sabbagh et al., 2010; Fookes et al., 2011; Lee et al., 2013
SPI-12	Variable (6–15)	*sspH2*	Actin polymerization, virulence in mice	*S. enterica* subsp. *enterica*	Morgan, 2007; Fookes et al., 2011; Tomljenovic-Berube et al., 2013
SPI-13	25	putative lyase, hydrolase, oxidase, and arylsulphatase regulator	Macrophage internalization, virulence in chickens, and mice	*S. enterica* subsp. *enterica* (NTS) *S. enterica* subsp. *arizonae* *S. enterica* subsp. *diarizonae* *S. enterica* subsp. *houtenae* (some)	Shah et al., 2005; Sabbagh et al., 2010; Fookes et al., 2011; Elder et al., 2016; Espinoza et al., 2017
SPI-14	9	*gpiAB*, putative acyl-CoA dehydrogenase	Chicken pathogenicity, epithelial invasion	*S. enterica* subsp. *enterica* (NTS) *S. enterica* subsp. *arizonae* *S. enterica* subsp. *houtenae* (some)	Shah et al., 2005, 2012; Fookes et al., 2011
SPI-15	6.5	Four putative ORFs	Unknown	*S. enterica* subsp. *enterica* serovar Typhi	Vernikos and Parkhill, 2006; Fookes et al., 2011
SPI-16	4.5	Bactoprenol glucosyl transferase and translocase (*gtrAB*)	LPS modification, seroconversion	*S. enterica* subsp. *enterica*	Vernikos and Parkhill, 2006; Fookes et al., 2011
SPI-17	5	Bactoprenol glucosyl transferase and translocase (*gtrAB*)	LPS modification, seroconversion	*S. enterica* subsp. *enterica*	Vernikos and Parkhill, 2006; Fookes et al., 2011
SPI-18	2.3	*hlyE* hemolysin, *taiA* invasion-associated protein	Epithelial invasion	*S. enterica* subsp. *enterica* *S. enterica* subsp. *diarizonae*	Den Bakker et al., 2011; Fookes et al., 2011
SPI-19	45	T6SS	Intramacrophage survival, chicken colonization	*S. enterica* subsp. *enterica*	Fookes et al., 2011; Blondel et al., 2013; Pezoa et al., 2014
SPI-20	34	T6SS	Unknown	*S. enterica* subsp. *arizonae*	Blondel et al., 2009, 2013; Fookes et al., 2011
SPI-21	55	T6SS	Unknown	*S. enterica* subsp. *arizonae*	Blondel et al., 2009, 2013; Fookes et al., 2011
SPI-22	20	T6SS	Unknown	*S. bongori*	Fookes et al., 2011
SPI-23	37	T3SS effectors (*sanA, chlR, shaU*, and *dumE*)	Host cell adherence and invasion, invasion of pig epithelial cells	*S. enterica* subsp. *enterica*	Hayward et al., 2013, 2014
SPI-24/CS54	25	Outer membrane protein (*shdA, sivH, ratAB, sinI*, and *potR*)	Fibronectin binding, murine intestinal colonization, and intramacrophage survival	*S. enterica* subsp. *enterica*	Kingsley et al., 2003; Sabbagh et al., 2010


*^*1*^SPI-10 was initially defined in *S*. Typhi and was thought to be unique to *S*. Typhi (Sabbagh et al., 2010), although other studies have reported the presence of SPI-10 genes in NTS serovars as well (Den Bakker et al., 2011; Desai et al., 2013). ^*2*^In some cases the size varies between serovars depending on the number of genes encoded. ^*3*^“some” indicates that not all serovars, or strains within a serovar, carry a given gene(s). ^*4*^Distribution based on screening of few representative strains, may not be representative of the entire subspecies as comprehensive studies for all serovars have not been conducted. For SPIs 5 and 11 no data were available for testing in subspecies other than *enterica.*^*5*^“Partial” indicates that strains tested in the labeled subspecies encoded part of the SPI, but not the full SPI.*

While SPIs are widespread among a number of *S. enterica* subsp. *enterica* serovars, some SPIs have been associated with select serovars, and are proposed to provide fitness advantages for these serovars. For example, SPI-7, encoding the Vi capsule, although classically thought to occur exclusively in *S*. Typhi (Faucher et al., 2005), has been found in strains of the NTS serovar Dublin as well (Morris et al., 2003; Pickard et al., 2003). It is interesting to note that both of these serovars are associated with invasive disease in humans. While SPI-11 is widespread among *S. enterica* subsp. *enterica* (Table 1), some serovars also encode genes for the typhoid toxin at this locus (Den Bakker et al., 2011). The presence of *cdtB* (a typhoid toxin gene) has been reported to be associated with significantly higher rates of invasive disease (Rodriguez-Rivera et al., 2015). Examples of SPIs associated with the ability to colonize a specific host have also been proposed. Mutation analyses of the T6SS encoded in SPI-19 in *S*. Gallinarum revealed a role for SPI-19 in the ability to colonize chickens (Blondel et al., 2010). However, introduction of SPI-19 (cloned from *S*. Gallinarum) in *S*. Enteritidis negatively impacted this serovar’s ability to colonize chickens (Blondel et al., 2009), therefore suggesting that, depending on the serovar, this SPI may provide a fitness advantage for some serovars, but not for others.

### Toxins

Exotoxins constitute toxins that are secreted. Originally identified in serotype Typhi, the typhoid toxin (or *Salmonella* cytolethal distending toxin) is a genotoxin that has recently been identified in at least 41 NTS serovars as well (Den Bakker et al., 2011), however, it is not found in serovars Typhimurium, Enteritidis, and Newport, which cause many of the clinical infections in the United States, and worldwide. Experiments with *S*. Typhi have revealed a putative role for this toxin in fine-tuning the host response to infection by targeting specific cell types such as endothelial cells in the brain, and immune cells (Yang et al., 2018). Injection of purified typhoid toxin has been shown to recapitulate some signs of typhoid fever in a mouse model (Song et al., 2013). In NTS, this toxin has been shown to contribute to systemic host colonization by *S*. Javiana (Miller et al., 2018).

The *spv* operon, primarily present on the *Salmonella* virulence plasmid (described below), encodes a toxin that mediates ADP-ribosylation of actin and is implicated in host cell cytoskeletal rearrangements, and finally, apoptosis of the host cell (Libby et al., 1997, 2000; Otto et al., 2000; Lesnick and Guiney, 2001). The *spv* locus has also been reported to be chromosomally encoded for some isolates in subspecies II, IIIa, IV, and VII (Boyd and Hartl, 1998). SpvC has been shown to contribute to virulence, due to its phosphothreonine lyase activity, which inhibits host MAP kinases (Guiney and Fierer, 2011). SpvB and SpvC are only found in a select number of serovars (see Table 2). Isolates encoding the *spv* operon are proposed to have enhanced virulence, and are often associated with invasive disease (Libby et al., 1997; Guiney and Fierer, 2011), as *spv* genes appear to play an important role in suppression of the innate immune response by attenuating the intestinal inflammatory response (Haneda et al., 2012).

**Table 2:** Selected nontyphoidal and paratyphoidal virulence plasmids.

Serovar	Plasmid name	Size (kb)
Abortusovis	pSAV	50–67
Abortusequi	pSTV	95
Choleraesuis	pSCV	50–110
Dublin	pSDV	80
Enteritidis	pSEV	60
Gallinarum/Pullorum	pSPV	85
Paratyphi C	pSPCV	55
Sendai	pSSV	285
Typhimurium	pSTV/pSLT	95


*Virulence plasmids are listed with their corresponding name and size in kilobase pairs (kb).*

The ArtAB toxin (for ADP-ribosylating toxin) is encoded by *S*. Typhimurium DT-104 strains (Saitoh et al., 2005) as well as multiple other NTS serovars (Rodriguez-Rivera et al., 2015). The active subunit, ArtA, ADP-ribosylates host G proteins (Uchida et al., 2009), while ArtB forms the pentameric binding subunit (Tamamura et al., 2017). ArtA and ArtB share homology with the active subunit (S1) and one of the B monomers (S2) of the binding subunit of the pertussis toxin, respectively (Tamamura et al., 2017). *In vitro*, the ArtAB toxin results in increased production of cAMP in RAW 264.7 cells, and a cell clustering phenotype in CHO cells (Saitoh et al., 2005; Tamamura et al., 2017). *In vivo*, BALB/c mice injected with 0.5–2 μg of purified ArtAB have significantly higher insulin secretion, and a reduced survival rate compared to mice injected with heat-killed toxin (Saitoh et al., 2005; Tamamura et al., 2017).

Several reports describe heat-labile, trypsin-sensitive cytotoxins produced by several NTS serovars. In some instances, cytotoxic activity is associated with the outer membrane. Cytotoxin production has been reported for extracts of serovars Braenderup, Choleraesuis, Enteritidis, Indiana, Nchanga, Saintpaul, Typhimurium, and Virchow (Reitmeyer et al., 1986; Ashkenazi et al., 1988; Kita et al., 1993; Malik et al., 1996). Considerable work has been conducted to characterize toxic activity in extracts and culture filtrates of multiple serovars (Peterson, 1980; Houston et al., 1981; Gemmell, 1984; Hariharan et al., 1986; Chopra et al., 1987; Khurana et al., 1991; Chary et al., 1993). One gene implicated in the observed cytotoxic activity, *stn*, has been proposed, although contrasting results about its actual involvement in cytotoxic activity exist, as its protein product has also been implicated in bacterial membrane integrity (Nakano et al., 2012).

### Flagella

Most NTS are capable of expressing flagella, which confer motility (Fàbrega and Vila, 2013; Rivera-Chavez and Baumler, 2015). An important exception is serovar Gallinarum biovars Gallinarum and Pullorum, which do not express phase 1 or phase 2 flagella and are therefore non-motile (Foley et al., 2013). Flagella synthesis, assembly, and maintenance requires >50 genes (Bonifield and Hughes, 2003). However, the antigenic subunit, flagellin, is encoded by three genes, *fliC* (phase 1), *fljB* (phase 2), and *flpA* (phase 3; rare and often plasmid-encoded) (McQuiston et al., 2004). For most NTS serovars, 5-10 flagella of peritrichous organization may be observed (Van Asten and Van Dijk, 2005). *Salmonella* employ phase variation, a reversible genetic rearrangement, to switch between expression of *fliC* and *fljB*, a mechanism that is utilized by a number of important bacterial pathogens (Silverman et al., 1979; Bonifield and Hughes, 2003; Garcia-Pastor et al., 2018).

While flagella aid *Salmonella* in migrating toward host epithelial layers, and thus are important virulence determinants, they are also potent inducers of the host innate immune response (Dos Santos et al., 2018). Flagella have also been shown to allow *Salmonella* to taxi toward the host-derived nitrate and tetrathionate, which are used as alternate terminal electron acceptors (Rivera-Chavez and Baumler, 2015).

Although there is no obvious evidence linking specific flagellar antigens to differences in virulence or host-adaptations, regulation of flagella upon infection has been established as a mechanism to reduce or prevent activation of a host immune response. For example, in *S*. Typhimurium, flagellar expression is down-regulated 50–100 fold during infection of RAW 264.7 murine macrophage cells (Srikumar et al., 2015). Furthermore, Spöring et al. (2018) showed that this downregulation is a response to alterations of the cell envelope as a result of cell envelope stress (Spöring et al., 2018). Studies of flagellar regulation in other NTS serovars may reveal key differences in expression, and provide additional roles by which NTS serovars are able to either intentionally trigger an inflammatory immune response, which has been associated with providing a metabolic niche allowing NTS serovars to propagate in the intestine (Rivera-Chavez and Baumler, 2015), or to evade killing by immune cells.

### Fimbriae (Pili)

Fimbriae are thin appendages that aid in attachment and adhesion, and are produced by a number of Gram-negative and Gram-positive bacteria. Phenotypic and genomic analyses have identified 39 putative fimbrial operons in *Salmonella.* Of these, the *agf* operon is found among isolates of both *S. enterica* and *S. bongori*, and encodes the nucleator-dependent curli fimbriae, which are thin, aggregative fimbriae that may aid in bacterial adhesion and invasion (Van Asten and Van Dijk, 2005; Yue et al., 2012). The *bfp* and *pil* operons encode type IV fimbriae; the latter operon is found on SPI-7, and its presence is therefore restricted to *S*. Typhi, Paratyphi C, and NTS serovar Dublin (Morris et al., 2003; Van Asten and Van Dijk, 2005). The remaining 36 fimbrial operons encode chaperone-usher-dependent fimbrial pathways (Clouthier et al., 1994; Van Asten and Van Dijk, 2005; Yue et al., 2012). Of these, 27 have been identified in NTS, with typical serovars containing 5–14 fimbrial gene clusters (Yue et al., 2012). The *fim* operon is the only chaperone-usher-dependent fimbrial operon found in all *S. enterica* isolates (Van Asten and Van Dijk, 2005; Yue et al., 2012). While the majority of fimbrial genes are not expressed under standard laboratory culturing conditions, most are expressed *in vivo* during infection (Laniewski et al., 2017), suggesting a role in adhesion to different tissues, or co-regulation with SPI-2 genes or other genes that are expressed when *Salmonella* is inside a host cell. The occurrence of other operons in various NTS has been reviewed elsewhere (Van Asten and Van Dijk, 2005). Of interest, the *pef* (plasmid encoded fimbriae) operon is located on a subset of *Salmonella* virulence plasmids (Rychlik et al., 2006), and is discussed later in further detail.

Differences in fimbrial gene clusters exist among serovars, and have been proposed as an additional mediator for allowing some serovars to colonize and persist in different hosts/environments (Yue et al., 2012). The number of intact fimbrial gene clusters, as well as the type, varies among serovars. For example, Yue et al. (2012) demonstrated that host adapted/restricted serovars, such as Typhi, Dublin, Paratyphi A, Choleraesuis, and Gallinarum encode multiple non-functional fimbrial genes (Yue et al., 2012). In contrast, serovars demonstrating a broader host range (Typhimurium, Enteritidis, Montevideo, Newport, Tennessee, Kentucky, and others) have relatively few degraded fimbrial genes (Yue et al., 2012). Moreover, allelic variation of fimbrial genes has also been proposed as an adaptation associated with the colonization of certain hosts (Yue et al., 2015). For example, *S*. Newport isolated from bovine, porcine, equine, or avian hosts was more likely to have allelic variants A/A/A1 (representing allelic combinations of FimH/BcfD/StfH), while *S*. Newport isolated from humans and the environment had predominately B/B/B1 fimbrial alleles (De Masi et al., 2017). A similar observation was also reported for *S*. Typhimurium, where different alleles of FimH1 or FimH7 were significantly associated with adherence to different human and bovine intestinal epithelial cell lines (Yue et al., 2015). Furthermore, Yue et al. (2015) also demonstrated inter-serovar differences in the ability of *E. coli* strains expressing different *Salmonella* FimH alleles to adhere to host cell lines representing different mammalian species (Yue et al., 2015). For example, expression of porcine-associated *Salmonella* fimbrial alleles resulted in significantly higher proportions of recombinant *E. coli* bound to porcine epithelial cell types (Yue et al., 2015). Together, this suggests that maintenance of multiple different fimbrial genes, and allelic variants of these genes, represents a mechanism by which NTS serovars are able to adhere to a variety of surfaces, making them suited to many different environments.

### Virulence Plasmids

**FIGURE 5:**
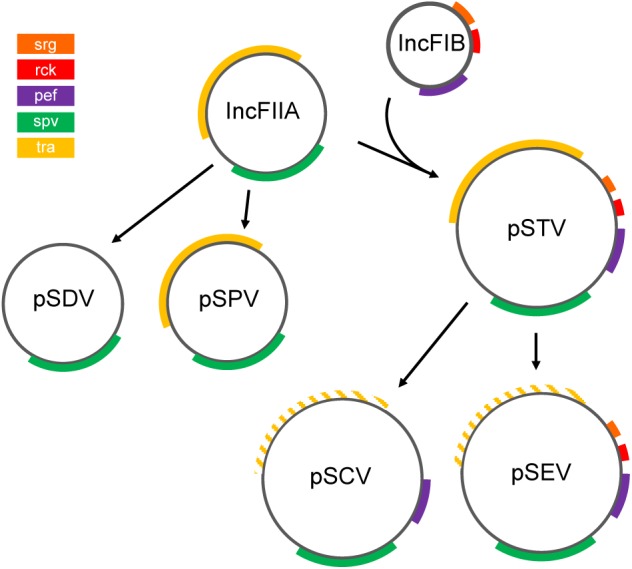
Proposed evolution of NTS virulence plasmids. The ancestral IncFIIA evolved into plasmids pSDV (Dublin) and pSPV (Gallinarum/Pullorum), or fused with replicon IncFIB in the pSTV (Typhimurium) lineage (Rychlik et al., 2006). pSCV (Choleraesuis) and pSEV (Enteritidis) are derived from pSTV (Chu and Chiu, 2006). Colored segments indicate gene clusters. Hatched lines indicate loss of function of the *tra* locus (Rotger and Casadesús, 1999). Although pSPV encodes *tra* genes, this plasmid is mobilized via the F-plasmid (Chu and Chiu, 2006). Plasmids and genes are not drawn to scale. This figure is inspired by the theories proposed by Rychlik et al. (2006) and others (Feng et al., 2012).

Nine NTS serovars have been described as harboring a low copy-number virulence plasmid. This plasmid varies among serovars, both in size and in genetic content (Table 2). Furthermore, carriage of the plasmid is not ubiquitous among all isolates of a given serovar (Chu and Chiu, 2006). Isolates that do carry the plasmid, though, generally exhibit increased virulence. The mediator of this increased virulence is the 7.8 kb *spvRABCD* (*Salmonella* plasmid virulence) operon, whose effectors alter the host cell cytoskeleton to enhance bacterial survival (Rotger and Casadesús, 1999). While *spv* is common to all *Salmonella* virulence plasmids, additional virulence factors or antimicrobial resistance genes may also be encoded (Figure 5).

The variation in content is attributed to the evolution of two distinct plasmid lineages, which have been characterized by sequence analysis, probe hybridization, and compatibility studies (Chu and Chiu, 2006). Serovars Paratyphi C (Liu et al., 2009), Sendai (Silva et al., 2017), Abortusequi (Akiba et al., 1999), and Abortusovis (Uzzau et al., 2000) also encode a virulence plasmid (Table 2), but the evolution of these plasmids is unclear.

The first lineage contains plasmids of *S*. Gallinarum (Pullorum; pSPV), and *S*. Dublin (pSDV) (Chu and Chiu, 2006). pSPV and pSDV harbor the replicon *repB*, which exhibits high similarity to the ancestral replicon IncFIIA (also called RepFIIA) (Rotger and Casadesús, 1999; Rychlik et al., 2006). It has been proposed that the ancestral IncFIIA replicon contained the *spv* and *tra* loci, as all *Salmonella* virulence plasmids encode variants of these genes, although the *tra* genes in the current pSPV and pSDV virulence plasmids are remnants of a bacterial conjugation system that has undergone variable degradation in several plasmid lineages (Rychlik et al., 2006). By virtue of the *tra* locus, pSPV may be mobilized in the presence of a F plasmid (Chu and Chiu, 2006; Rychlik et al., 2006), whereas pSDV lacks homology to the *tra* locus (Rotger and Casadesús, 1999), and cannot be mobilized (Rychlik et al., 2006), suggesting substantial deterioration of the *tra* locus in pSDV.

The plasmids of serovars Typhimurium (pSTV), Enteritidis (pSEV), and Choleraesuis (pSCV) compose the second lineage (Chu and Chiu, 2006), and contain not only the *repB* replicon, but also elements of a second replicon, *repC* (Rotger and Casadesús, 1999). *repC* exhibits similarity to the IncFIB replicon (Rotger and Casadesús, 1999), which likely encoded the virulence factors *pef, srgAB*, and *rck* (Rychlik et al., 2006). The *pef* operon, or plasmid-encoded fimbriae, is conserved in all three plasmids of this lineage (Rychlik et al., 2006). *pef-*encoded fimbriae mediate adhesion to cells within the small intestine, and exhibit preferential binding to certain host cells of various species (Baumler et al., 1996). The *srgA* gene product is important for the biogenesis of the *pef*-encoded fimbriae (Bouwman et al., 2003), yet this locus has been lost in pSCV (Rychlik et al., 2006), as has *rck* (Rychlik et al., 2006). *rck* has been shown to contribute to resistance to complement killing, one of the host’s innate immune responses (Rychlik et al., 2006). As in the first lineage the *tra* locus exhibits variable degradation among the plasmids pSTV, pSCV, and pSEV. The *tra* operon of pSTV renders that plasmid capable of conjugative transfer (Rychlik et al., 2006), yet in the *S*. Enteritidis plasmid (pSEV), and *S*. Choleraesuis plasmid (pSCV) lineages the *tra* locus has degraded and is no longer functional (Rotger and Casadesús, 1999; Rychlik et al., 2006). The degradation of *tra* in pSCV and pSEV may indicate that they descended from pSTV via deletions (Chu and Chiu, 2006).

## Disease Diversity-Nontyphoidal Salmonellosis Disease Severity Varies Significantly by Serovar

Although the complete host-pathogen relationship plays an important role in determining the resulting severity of salmonellosis (Figure 2), some NTS serovars excel at causing invasive human clinical infections that are reminiscent of the pathology exhibited by Typhi and paratyphoidal serovars of *S. enterica.* Invasive infections result from *Salmonella*’s successful ability to escape the gastrointestinal tract, and subsequently spread to other tissues. These infections are considered to be the most severe, often resulting in hospitalization. Most commonly, invasive salmonellosis is defined as isolation of *Salmonella* from a “sterile site,” usually blood, joint fluid, or cerebrospinal fluid (Jones et al., 2008). Global estimates of invasive nontyphoidal salmonellosis (regardless of serovar) range from 30 to 227 cases per 100,000 persons (Ao et al., 2015). With the exception of select NTS serovars, most invasive NTS infections are typically associated with individuals from so-called “high risk” populations (i.e.,<5 years old, >65 years old, having an immunodeficiency, or being pregnant). For example, invasive nontyphoidal salmonellosis is more frequently cited in Africa (Okoro et al., 2012; Ao et al., 2015) and Southeast Asia (Lan et al., 2016), and is associated with malnutrition, immunodeficiencies including HIV, and malaria co-infection (Scott et al., 2011; Feasey et al., 2012).

A study by Jones et al. (2008) found that, compared to *S*. Typhimurium infections, of which just 5.7% resulted in invasive disease, infections with serovars Choleraesuis (56.4%), Dublin (64.0%), Sandiego (18.9%), and Panama (18.0%) were associated with significantly higher rates of invasive disease among human clinical infections in the US (Jones et al., 2008). While the total number of salmonellosis cases contributed by these serovars is considerably lower than those of other serovars, the number of infections resulting in invasive disease is significantly higher, suggesting that either (i) most of these infections arise from individuals in high risk populations (i.e., only select sub-populations are susceptible to infection with these serovars, and when infection occurs it is often more severe), (ii) the majority of the cases are asymptomatic, and therefore only severe cases are reported, or (iii) these serovars have virulence factors or adaptations that make them inherently more invasive. While further research is needed to conclusively determine which components of the host-pathogen interaction specifically account for the observed invasive phenotype of these serovars, the current understanding is summarized below.

### NTS Serovars Associated With High Rates of Invasive Disease in Humans

*Salmonella enterica* subsp. *enterica* serovar Choleraesuis (*S*. Choleraesuis) is a host-adapted serovar, causing swine paratyphoidal disease (Chiu C.-H. et al., 2004; Pedersen et al., 2015). In humans, *S*. Choleraesuis often causes septicemia with minimal GI-tract inflammation (Chiu C.-H. et al., 2004), resulting in a disease more similar to typhoid fever (Sabbagh et al., 2010). This suggests that, similar to *S*. Typhi, the severity of the salmonellosis caused by this serovar results from successful evasion of host defenses in the gut, and thus failure of the immune system to detect and control *S*. Choleraesuis early in the infection. In support of this, most human clinical cases of *S*. Choleraesuis occur in patients with a pre-existing health condition, such as an immunosuppressive condition (Wang et al., 2006), or other chronic disease (Chiu S. et al., 2004). Like other serovars that are frequently associated with invasive human disease, *S*. Choleraesuis encodes a T6SS, which plays a role in virulence *in vivo* (Schroll et al., 2019). *S*. Choleraesuis strain SC-B67, originally isolated from a human patient with sepsis, has a high proportion of pseudogenes, most of which arose from SNPs resulting in premature stop codons in genes related to metabolism, fimbriae, and the chemotactic response (Chiu et al., 2005). Inactivation of several ancestral genes may limit the adaptability of *S*. Choleraesuis in several niches including animal hosts and environmental sites, which would account for the lack of clinical cases in animals other than pigs and humans for this serovar. While *S*. Choleraesuis is rarely isolated from human clinical cases in the US [∼10–20 confirmed cases per year (Centers for Disease Control and Prevention [CDC], 2016)] and the EU (Pedersen et al., 2015), cases of *S*. Choleraesuis are relatively more common in Southeast Asia such as in Taiwan and Thailand, although control efforts have been very successful at reducing the incidence of *S*. Choleraesuis infections in these areas (Hendriksen et al., 2009; Hendriksen et al., 2011).

*Salmonella enterica* subsp. *enterica* serovar Dublin (*S*. Dublin) is a bovine host-adapted serovar (Langridge et al., 2015). Genetic analyses have shown that *S*. Dublin shares a common ancestor with another host-adapted serovar, *S*. Gallinarum (Langridge et al., 2015), although *S.* Dublin has substantially fewer pseudogenes (Langridge et al., 2015). *S*. Dublin encodes two T6SSs located in SPI-6 and SPI-19 (Mohammed et al., 2017). In the United States, confirmed human clinical cases of *S*. Dublin salmonellosis contribute ∼150 reported cases per year (Centers for Disease Control and Prevention [CDC], 2016), with the majority of *S*. Dublin infections occurring in adults (median age 55 years, compared to median age of 23 years for infections with all other *Salmonella*) (Harvey et al., 2017). This suggests that either the incidence of exposure to *S*. Dublin is higher among older individuals, or *S*. Dublin is asymptomatic in younger individuals, but causes clinical disease when it encounters a host with a weakened immune system, as is common for other opportunistic pathogens. While *S*. Dublin infections in humans are significantly more likely to result in hospitalization, invasive disease, prolonged hospital stays, and an increased likelihood of death (Harvey et al., 2017), the true incidence of exposure is unknown, and is likely much higher than the number of clinical infections reported each year. Nevertheless, it is intriguing that this serovar, when successful in colonizing humans, often results in invasive disease.

Infections with *S. enterica* subsp. *enterica* serovar Sandiego (*S*. Sandiego) and *S. enterica* subsp. *enterica* serovar Panama (*S*. Panama) are also associated with high rates of invasive disease (Jones et al., 2008; Crump et al., 2011). In contrast to *S*. Dublin and *S*. Choleraesuis, these serovars have not been characterized as being host-adapted. *S*. Sandiego has been linked to several outbreaks from handling small turtles (Walters et al., 2015), and has also been isolated from livestock including pigs (Oliveira et al., 2002), goats (El Tom et al., 1999), and cows (Boqvist and Vågsholm, 2005). *S*. Panama has been isolated from pigs, poultry, cows, and goats (Cordano and Virgilio, 1996). Both *S*. Panama and *S*. Sandiego encode *S*. Typhi-associated SPI-18 genes *hlyE* and *taiA*, and the typhoid toxin genes (*cdtB, pltA*, and *pltB*) (Den Bakker et al., 2011), although the role that these genes, and their corresponding gene products, play in invasive infections involving these serovars has not been assessed. Unlike *S*. Choleraesuis and *S*. Dublin, infections with *S*. Panama and *S*. Sandiego are more commonly diagnosed among children <5 years old (Boore et al., 2015). Therefore, either environmental exposure to *S*. Panama and *S*. Sandiego is more common in young children, perhaps as a result of animal contact, or these serovars possess virulence factors that enable them to successfully cause disease in young children, but not as frequently in adults, as is the case for serovars Choleraesuis and Dublin.

### NTS Serovars Associated With Reduced Rates of Human Clinical Disease: Evidence of Loss of Virulence

In contrast to NTS serovars associated with high rates of invasive human clinical disease, there also exist a number of serovars that are commonly isolated from agricultural reservoirs, but which cause a disproportionately low number of human clinical cases. The primary reasons for this include (i) effective kill steps such as heating or other inactivation techniques for contaminated food commodities (i.e., cooking of meat or pasteurization of milk), (ii) mutations in select serovars that effectively reduce their ability to cause disease in select hosts, and (iii) genetic adaptations that allow the serovar to colonize an animal host, but do not impart a fitness advantage allowing for disease manifestation in humans.

*S*. Cerro is one of the most frequently isolated serovars among dairy clinical isolates (Tewari et al., 2012; Valenzuela et al., 2017), although this serovar is responsible for just ∼30 reported human clinical cases per year in the United States (Centers for Disease Control and Prevention [CDC], 2016). In 2016, *S*. Cerro was the 4th most common clinical isolate among animal clinical cases reported to the USDA, and represented the 2nd most common serovar reported among clinical cases in cattle (Morningstar-Shaw et al., 2016). Despite *S*. Cerro being detected in bulk tank raw milk samples, outbreaks from *Salmonella*-contamination of raw milk in the United States usually involve serovars Typhimurium (Mungai et al., 2015), Montevideo, and Newport (Robinson et al., 2014), and the cow-adapted serovar Dublin (Harvey et al., 2017; Vignaud et al., 2017). *S*. Cerro isolates have a premature stop-codon in *sopA* (Rodriguez-Rivera et al., 2014; Kovac et al., 2017), a virulence factor shown to play an important role in *Salmonella* entry into host cells (Raffatellu et al., 2005). Indeed, *S*. Cerro isolates have a significantly lower rate of invasion in the Caco-2 cell line, compared to *S*. Typhimurium and *S*. Newport strains in a standard gentamicin protection assay (Rodriguez-Rivera et al., 2014). The comprehensive set of reasons behind the discrepant ability of *S*. Cerro to colonize and amplify within dairy cattle, and its low likelihood of causing clinical disease in both humans and most animals, remains unknown.

In the United States, *S*. Kentucky represents just 0.1% of reported human clinical cases of salmonellosis (Centers for Disease Control and Prevention [CDC], 2016), yet it is the most commonly isolated serovar from broiler chickens in the United States (United States Department of Agriculture [USDA], 2014). Although experimental evidence suggests that *S*. Kentucky may persist for longer periods of time in chickens than *S*. Typhimurium, the reasons why *S*. Kentucky is infrequently associated with human clinical cases of salmonellosis have yet to be confirmed (Cheng et al., 2015). One possibility for the lack of human clinical cases is that *S*. Kentucky isolates lack the virulence genes *grvA, sseI, sopE*, and *sodCI* (Beutlich et al., 2011; Cheng et al., 2015), which may play a role in human disease but are not necessary for colonizing chickens. Another study by Tasmin et al. (2017) using a phenotypic array found that *S*. Kentucky ST152 strain SK222_32B differed significantly in its ability to utilize a number of common metabolites, namely 1,2-propanediol (Tasmin et al., 2017), which has previously been cited for enabling *S*. Typhimurium to successfully outcompete the resident microbiota in the mammalian gut (Faber et al., 2017). *S*. Kentucky SK222_32B also failed to replicate in macrophages, implying that *S*. Kentucky has an impaired ability to resist immune-killing, possibly as a result of its lacking super oxide dismutase (*sodCI*) (Tasmin et al., 2017). Indeed, other studies have shown that, when exposed to media at pH 2.5, *S*. Kentucky was more sensitive to acid stress than serovars Enteritidis, Hadar, Mbandaka, Senftenberg, Typhimurium, and Worthington (Joerger et al., 2009). In contrast, human clinical cases caused by *S*. Kentucky are increasing in Europe. Interestingly, *S*. Kentucky type ST198 is more commonly isolated in human clinical cases (primarily in Europe and Northern Africa), but *S*. Kentucky ST152 is more commonly isolated from poultry (Le Hello et al., 2013; Haley et al., 2016). While studies suggest that ciprofloxacin resistance encoded by *S*. Kentucky ST198 explains its sudden expansion in human clinical cases (Le Hello et al., 2011, 2013), future studies examining the genetic and metabolic differences between the chicken-associated (ST152) and human associated (ST198) STs may reveal more conclusive evidence for why certain *S*. Kentucky STs are associated with different hosts.

Understanding why certain serovars are able to asymptomatically colonize a host represents an important gap in our current understanding of nontyphoidal salmonellosis. In the case of *S*. Cerro, understanding the importance of mutations enabling this serovar to frequently colonize cattle, but rarely cause clinical symptoms in humans represents a key gap in our understanding of NTS virulence. For *S*. Kentucky, increases in human clinical infections involving a specific sequence type suggest either a new route of transmission, or possibly a novel adaptation of ST198 that may be absent in other *S*. Kentucky STs, which might be an important model for how other serovars that are frequently associated with animal reservoirs can adapt to cause human clinical disease.

## Adapting to the Situation – Foodborne, Zoonotic, and Environmental Sources of NTS Serovars

While the vast majority of NTS cases are foodborne, infections resulting from animal contact and environmental exposures have been reported. Owing to their ability to successfully survive in a number of different environments, NTS serovars have adapted to achieve a number of different routes of transmission to cause human clinical infections. Traceback investigations aimed at linking a *Salmonella* isolate to its source represent a persistent challenge. Further complicating this, the majority of infections represent sporadic incidents, which cannot be linked to a common source. Here we review the foodborne, animal-contact, and environmental sources of NTS salmonellosis.

### Foodborne Nontyphoidal Salmonellosis

**FIGURE 6:**
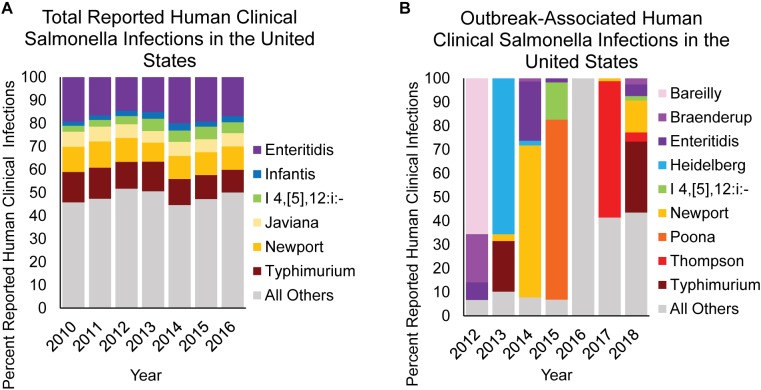
NTS causing overall vs. confirmed outbreak-associated infections in humans. The contribution of select NTS serovars to **(A)** overall human clinical infections vs. **(B)** human clinical infections confirmed from a foodborne outbreak are shown as percent infection for a given category. Data are modified from Centers for Disease Control and Prevention [CDC] (2016, 2018).

In the United States, approximately 94% of domestically acquired salmonellosis cases are acquired through the consumption of contaminated food (Scallan et al., 2011), with most representing sporadic cases (Ebel et al., 2016). In fact, it has been reported that just 5.9% of NTS infections in the United States are linked to an outbreak (Ebel et al., 2016). This is best represented by comparing the relative contribution of reported NTS serovars isolated from human clinical infections (Figure 6A) with the serovars causing outbreaks traced back to a single food item (Figure 6B). Data compiled between 2012 and 2018 show that in the United States, the primary NTS serovars associated with outbreaks vary significantly from year to year, and are often the result of a few large outbreaks (Figure 6B). The overall trend in human clinical infections remains constant however, as infections reported between 2010 and 2016 in the United States show very little fluctuation by serovar, with the top 6 serovars accounting for ∼50% of all infections each year. Therefore, the accumulation of NTS sporadic infections is likely the result of low-level *Salmonella* contamination from a wide range of foods, primarily by serovars Typhimurium, Newport, Enteritidis, Javiana, Infantis, and I 4,[5],12:i:-, and not due to large outbreaks involving these serovars.

**FIGURE 7:**
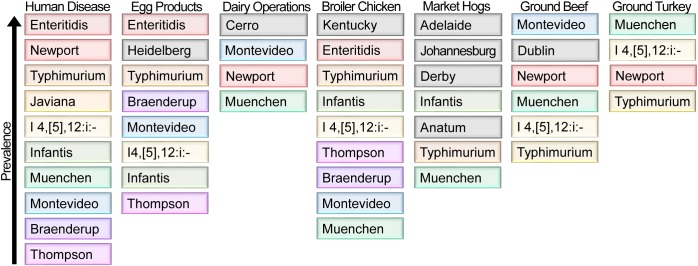
NTS serovar prevalence from reported human clinical cases of salmonellosis and surveillance of different animal food sources. The ten serovars most frequently identified in human disease (colored) are listed in order of their prevalence for each of the indicated sources (United States Department of Agriculture [USDA], 2014, 2017; Centers for Disease Control and Prevention [CDC], 2016; Sonnier et al., 2018). When a serovar represents ≥10% of *Salmonella* identified from a source other than humans, it is shown in gray under that source.

Each food commodity presents its own challenges, including differences in pH, available water, temperature, nutrient composition, osmolarity, and the presence of antimicrobial compounds; combined these represent unique environmental stresses to which *Salmonella* must adapt. An analysis by the CDC of the food commodities most frequently associated with outbreaks of salmonellosis by serovar reveals that for certain food products, there is a strong association between select serovars and food commodities of animal origin (Jackson et al., 2013). For example, for serovars Enteritidis, Heidelberg, I 4,[5],12:i:-, and Hadar, the majority of outbreaks are associated with poultry (either chicken or turkey meat, or eggs), while serovars Infantis, Montevideo, and Uganda are more commonly associated with outbreaks linked to consumption of pork and beef (Jackson et al., 2013). This suggests that these serovars possess genetic and phenotypic traits that promote their ability to adapt to these environments. The USDA conducts routine surveillance in the United States to monitor retail-bound meat (United States Department of Agriculture [USDA], 2014), egg products (United States Department of Agriculture [USDA], 2017), and raw dairy foods (Sonnier et al., 2018) for contamination by NTS. An interesting observation is that the serovars most frequently isolated from these high-risk food sources do not mirror those predominantly responsible for infection in humans (Figure 7). This is readily apparent from the frequent isolation of *S*. Kentucky and *S*. Cerro from poultry meat products and dairy cows, respectively, which does not result in an equivalent predominance among human clinical infections (Figure 7).

A number of produce (fruits and vegetables) outbreaks have also occurred (Hanning et al., 2009), demonstrating NTS serovars’ ability to survive in acidic and dry environments, as well as at a wide range of temperatures associated with the produce production chain. For example, tomatoes have been associated with several outbreaks involving serovars Montevideo, Newport, Braenderup, and Javiana (Hanning et al., 2009). Shi et al. (2007) observed differences among serovars that were able to grow on ripened tomatoes, likely due to the high acidity associated with this fruit (Shi et al., 2007). Differences in acid tolerance among NTS serovars, may therefore represent one mechanism allowing some serovars to contaminate and grow in select food commodities.

### Zoonotic Transmission of *Salmonella*

**FIGURE 8:**
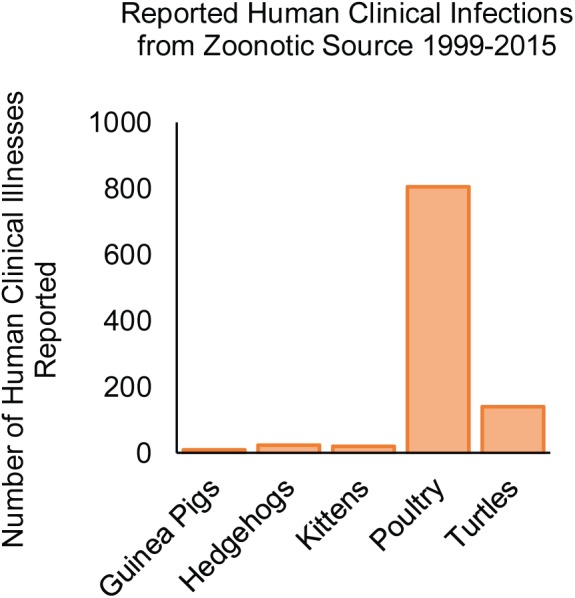
NTS serovars associated with zoonotic infections in the United States from 1999 to 2015. The number of human clinical infections resulting from animal contact is shown according to the source of the infection (Centers for Disease Control and Prevention [CDC], 2001; Wright et al., 2005; Loharikar et al., 2012; Bartholomew et al., 2014; Nakao et al., 2015; Anderson et al., 2016, 2017; Gambino-Shirley et al., 2018).

Several reports implicate a role for animal contact in human infection with NTS serovars. Not surprisingly, handling of chicks is responsible for a considerable number of salmonellosis cases (Figure 8), in which serovars Typhimurium, Johannesburg, Braenderup, Thompson, and Montevideo are frequently implicated with human outbreaks of salmonellosis (Loharikar et al., 2012; Pabilonia et al., 2014; Habing et al., 2015; Nakao et al., 2015; Anderson et al., 2016; Sharma et al., 2018). Zoonotic infection may also result from exposure to infected companion animals (kittens, guinea pigs, hedgehogs, and turtles) (Figure 8; Centers for Disease Control and Prevention [CDC], 2001; Wright et al., 2005; Bartholomew et al., 2014; Anderson et al., 2017; Gambino-Shirley et al., 2018). Interestingly, NTS infections arising from contact with mammals have been largely linked to serovars Typhimurium and Enteritidis (Hoelzer et al., 2011) (the two serovars causing the majority of foodborne salmonellosis in humans), while zoonoses contracted from turtles included infection with serovars Sandiego, Poona, and Pomona (Gambino-Shirley et al., 2018), which infrequently cause human clinical cases.

### Environmental Transmission of *Salmonella*

Although less common, nontyphoidal serovars have been isolated from soil, water, contaminated floors, carts, and other equipment-related sources (Cummings et al., 2014; Strawn et al., 2014; Liu et al., 2018). Environmental sources of NTS serovars often arise from the introduction of NTS-contaminated animal feces, either via contaminated water used to irrigate crops, or from direct contact with feces from the *Salmonella*-secreting animal. In the case of several teaching hospitals, nosocomial transmission has been confirmed between animals, including different species, implicating the ability of NTS serovars to survive and transit from different environmental sources, to a susceptible host (Cummings et al., 2014). Furthermore, albeit rare, human-human cases of NTS transmission have also been documented (Kariuki et al., 2006).

Outbreaks of foodborne salmonellosis have also been documented in which biofilms harboring NTS serovars were linked to contamination of food products. In 2004 and 2005, two outbreaks involving *S*. Agona in powdered infant formula were traced back to environmental contamination in the production plant where the formula was produced (Brouard et al., 2007). *S*. Agona has also been linked to two outbreaks in dry cereal, in 1998 and 2008 (Russo et al., 2013), resulting from *S*. Agona persistence in the food processing plant. Importantly, these outbreaks highlight the potential for NTS serovars to persist in food processing plants for extended periods of time, and then contaminate food products.

Depending on the environment, transmission of NTS serovars from the environment onto a food surface represents an important, yet indirect, mechanism of transmission for a number of food commodities. While adaptations that allow NTS serovars to persist in different environments, such as *Salmonella*’s ability to survive in dry foods/environments, are largely dependent on the specific food (i.e., fat content and moisture content, etc.), there are significant differences in the abilities of different serovars to survive in foods.

## Reducing NTS Incidence Will Require a Diversified Approach

The broad host-range displayed by a number of NTS serovars represents a key challenge in reducing nontyphoidal salmonellosis, as interventions aimed at targeting a single reservoir are often unsuccessful at eliminating *Salmonella* from other reservoirs, many of which are unknown. Furthermore, successful control of one serovar has historically been associated with increases in the number of human clinical cases and outbreaks caused by other serovars due to the vacancy of a previously occupied niche. For example, targeted control efforts aimed at eliminating *S*. Gallinarum (biovars Gallinarum and Pullorum) infections among chickens paralleled the sudden increase in *S*. Enteritidis, which is able to colonize chickens without causing overt clinical disease (Baumler et al., 2000). Therefore, successful control of one serovar in a given niche may enable the expansion of a new serovar. Complicating this, little is known about the natural reservoirs of the less-studied NTS serovars.

The vast majority of what is known about the virulence factors and mechanisms of NTS serovars is derived from experiments performed with NTS serovar Typhimurium, which have provided beneficial data that have expanded our understanding of how *Salmonella* cause disease. However, the genetic differences among serovars suggest that a number of mechanisms are serovar-specific, and therefore, improved approaches should consider the importance of using specific serovars (Jones et al., 2008; Suez et al., 2013). Identifying what makes certain serovars better adapted to persist in different environments also represents a key piece in resolving the NTS-contamination puzzle, as many serovars have yet to be associated with their reservoir(s).

The collective effort to reduce-foodborne nontyphoidal salmonellosis will require a collaboration of both laboratory science, generating experimental evidence about how diverse NTS serovars are able to overcome unfavorable environmental conditions to establish infection, and public health authorities utilizing novel methods for enhanced outbreak detection and source tracking. Furthermore, increases in the diversity and number of publicly available WGS data will enable the use of genetic tools (i.e., CRISPR, Lambda-Red mutagenesis, etc.) necessary to carry out genetic and phenotypic analyses among non-Typhimurium NTS serovars, allowing for the development of novel control and treatment strategies that will aid in reducing the overall morbidity and mortality associated with NTS on a global level.

As foodborne transmission represents the most common route of infection with NTS serovars, risk-based research approaches aimed at identifying and targeting specific serovars likely to contaminate a given food product represents an attractive approach to reducing human clinical cases. For example, the synthesis of data regarding contamination of a specific food commodity, such as *S*. Enteritidis and eggs, can be used to identify key sources of contamination so that research and regulatory initiatives may be focused to control contamination with specific serovars in these niches. Alternatively, assessments based on disease severity may be used to inform new regulatory decisions, such as imposing more stringent limits for some serovars (e.g., those associated with high rates of invasive disease), or relaxed limits for less virulent serovars (e.g., Cerro) or for specific strains showing virulence-attenuating mutations.

## Conclusion

While the global burden of nontyphoidal salmonellosis remains as one of the key challenges, novel methods for studying *Salmonella* have changed the way that the research community detects, investigates, and prevents salmonellosis. Reflecting the diversity of virulence factors, genetic and phenotypic adaptations among the more than 2,600 serovars, reducing contamination with NTS serovars represents a multi-faceted challenge that will require collaborations of industry, government, and academia to achieve the ultimate goal of reducing NTS human clinical infections worldwide.

## Author Contributions

RC, CE, and MW conceived and revised the manuscript.

## Conflict of Interest Statement

The authors declare that the research was conducted in the absence of any commercial or financial relationships that could be construed as a potential conflict of interest.
